# The Anti-Diabetic Drug Metformin Protects against Chemotherapy-Induced Peripheral Neuropathy in a Mouse Model

**DOI:** 10.1371/journal.pone.0100701

**Published:** 2014-06-23

**Authors:** Qi-Liang Mao-Ying, Annemieke Kavelaars, Karen Krukowski, Xiao-Jiao Huo, Wenjun Zhou, Theodore J. Price, Charles Cleeland, Cobi J. Heijnen

**Affiliations:** 1 Neuroimmunology Laboratory, Department of Symptom Research, University of Texas M.D. Anderson Cancer Center, Houston, Texas, United States of America; 2 Department of Integrative Medicine and Neurobiology, State Key Laboratory of Medical Neurobiology, School of Basic Medical Sciences, Fudan University, Shanghai, People's Republic of China; 3 School of Behavioral and Brain Sciences, University of Texas at Dallas, Richardson, Texas, United States of America; 4 Department of Symptom Research, University of Texas M.D. Anderson Cancer Center, Houston, Texas, United States of America; University of South California, United States of America

## Abstract

Chemotherapy-induced peripheral neuropathy (CIPN) characterized by loss of sensory sensitivity and pain in hands and feet is the major dose-limiting toxicity of many chemotherapeutics. At present, there are no FDA-approved treatments for CIPN. The anti-diabetic drug metformin is the most widely used prescription drug in the world and improves glycemic control in diabetes patients. There is some evidence that metformin enhances the efficacy of cancer treatment. The aim of this study was to test the hypothesis that metformin protects against chemotherapy-induced neuropathic pain and sensory deficits. Mice were treated with cisplatin together with metformin or saline. Cisplatin induced increased sensitivity to mechanical stimulation (mechanical allodynia) as measured using the von Frey test. Co-administration of metformin almost completely prevented the cisplatin-induced mechanical allodynia. Co-administration of metformin also prevented paclitaxel-induced mechanical allodynia. The capacity of the mice to detect an adhesive patch on their hind paw was used as a novel indicator of chemotherapy-induced sensory deficits. Co-administration of metformin prevented the cisplatin-induced increase in latency to detect the adhesive patch indicating that metformin prevents sensory deficits as well. Moreover, metformin prevented the reduction in density of intra-epidermal nerve fibers (IENFs) in the paw that develops as a result of cisplatin treatment. We conclude that metformin protects against pain and loss of tactile function in a mouse model of CIPN. The finding that metformin reduces loss of peripheral nerve endings indicates that mechanism underlying the beneficial effects of metformin includes a neuroprotective activity. Because metformin is widely used for treatment of type II diabetes, has a broad safety profile, and is currently being tested as an adjuvant drug in cancer treatment, clinical translation of these findings could be rapidly achieved.

## Introduction

Chemotherapy-induced peripheral neuropathy (CIPN), characterized by a glove-and-stocking distribution of sensory changes including dysesthesia, paraesthesia, and pain, occurs on average in 30-40% patients treated for cancer, but the incidence ranges widely among various studies and compounds from 10–100% [1], [2]. A deficit in touch detection that ranges from the pain area into the volar skin outside the area of perceived pain or sensory dysfunction has also been reported in patients treated with chemotherapy [2], [3]. In patients treated for cancer, the pain, numbness and tingling in hand and feet develop during treatment and frequently continue even after completion of therapy. Moreover, this peripheral neuropathy is a major dose-limiting adverse effect of multiple chemotherapeutic agents, including cisplatin (cis-diamminedichloroplatinmum) and paclitaxel. At present, there are no FDA-approved treatments for chemotherapy-induced neuropathy. Therefore, identification of drugs that provide effective prevention and/or treatment of chemotherapy-induced peripheral neuropathy would be a major step forward in improving treatment adherence and quality of life in patients treated with chemotherapeutics.

Metformin, a classic and widely used anti-diabetic drug, activates adenosine monophosphate-activated protein kinase (AMPK). Recent findings show that metformin reverses established mechanical allodynia in models of neuropathic pain induced by spinal nerve ligation in rats and spared nerve injury in mice [4], [5]. Moreover, metformin attenuates hyperexcitability in sensory neurons that develops following exposure to growth factors and cytokines that have previously been linked to chemotherapy-induced neuropathic pain [6]. In addition, metformin has neuroprotective effects in murine models of neurodegenerative disorders including diabetes-associated brain neurodegeneration [7], [8], ethanol-induced neuronal apoptosis [9] and experimental stroke [10]. There is evidence that metformin enhances the efficacy of anti-cancer treatment [11], [12] an effect that has been widely attributed to the drug's action on AMPK. However, whether metformin protects against cisplatin-induced neuropathy has not been tested.

Intraepidermal nerve fibers (IENFs) are free nerve ending arising from unmyelinated and thinly myelinated sensory neurons within the dermis and are important for transmission of peripheral pain [13]. In humans treated with chemotherapy, the density of epidermal nerve fibers in the fingertip, palm and forearm are either absent or reduced, indicating damage to distal nerves [1]. Similarly, in rats and mice receiving repeated treatment with platinum-based chemotherapeutic agents a significant reduction in IENF density is observed in the foot pad [13], [14], [15]. In 2011, J. Boyette-Davis et al. reported that minocycline treatment effectively prevented IENF loss as well as mechanical allodynia in oxaliplatin-treated rats [13]. This finding revealed that peripheral nerve damage leading to a reduction in IENFs may be involved in chemotherapy-induced neuropathic pain. In addition, IENFs are known to be important for normal tactile sensation [16], [17]. Hence, treatments that protect against chemotherapeutic-induced retraction and/or damage of IENFs may reduce the development of neuropathic pain and alleviate dysesthesias that are common dose limiting complaints in patients.

Here we have tested the hypothesis that metformin may protect against chemotherapy-induced neuropathic pain, dysesthesia and loss of IENFs in the hind paw of mice. This hypothesis is based on the notion that AMPK activation may lead to the production of direct and/or indirect neuroprotective and anti-hyperexcitability effects in the peripheral nervous system that would alleviate multiple facets of chemotherapy-induced sensory symptoms [18]. Our findings show that metformin is broadly effective in a preclinical mouse model suggesting that these findings may rapidly translate into transformative treatment strategies for the alleviation of these dose-limiting side effects.

## Materials and Methods

### Animals

C57Bl/6J mice at 12 weeks of age at the beginning of the experiment were used in this study. Mice were housed on a 12 h light/dark cycle with free access to food and water. All behaviors were observed during the dark phase of the light cycle with red light. All procedures were consistent with the National Institute of Health Guidelines for the Care and Use of Laboratory Animals and the Ethical Issues of the International Association for the Study of Pain [19] and were approved by the Institutional Animal Care and Use Committee of Texas A&M University (protocol number 12020).

### Drug treatments

After repeat exposure to the test environment and baseline measurements of behavior, C57Bl/6J mice were intraperitoneally (i.p.) treated with cisplatin (Sigma-Aldrich, St. Louis MI) daily at a dosage of 2.3 mg/kg for 5 days, followed by 5 days of rest, for two cycles with a total cumulative dose of 23 mg/kg cisplatin [19]. Control mice received an equivalent volume of saline. Metformin hydrochloride (Sigma-Aldrich) was freshly prepared in saline daily. Metformin (200 mg/kg) or saline was given i.p. for seven consecutive days, beginning 24 h prior to the first dose of cisplatin of each cycle or 24 h before the start of the second cisplatin cycle. A separate group of mice was treated with paclitaxel (i.p 10 mg/kg) every other day for two weeks. In this group, metformin (200 mg/kg i.p.) was given daily starting 24 h before the first dose of paclitaxel. On days when both cisplatin or paclitaxel and metformin were administered, metformin was given 30–60 min prior to cisplatin or paclitaxel. Lidocaine hydrochloride (Sigma-Aldrich; 5 µl 4% in saline) or saline was injected into the hind paw of control mice 10 minutes before the adhesive removal test.

### Von Frey test for mechanical allodynia

Mechanical allodynia was measured as the hind paw withdrawal response to von Frey hair stimulation using the up-and-down method as we described previously [20]. Mice were placed in a plastic cage (10×10×13 cm^3^) with a mesh floor for 30 min prior to testing. Subsequently, a series of von Frey hairs (0.02, 0.07, 0.16, 0.4, 0.6, 1.0 and 1.4 g) (Stoelting, Wood Dale, IL) were applied perpendicular to the mid-plantar surface of hind paw. A trial began with the application of the 0.16 g hair. A positive response was defined as a clear paw withdrawal or shaking. Whenever a positive response occurred, the next lower hair was applied, and whenever a negative response occurred, the next higher hair was applied. The testing consisted of five more stimuli after the first change in response occurred, and the pattern of response was converted to the 50% withdrawal threshold using the method described previously [21] and specified in the supporting information (File S1).

### Rotarod test for motor impairment

To control for the possibility that behavioral changes are due to the motor impairment in cisplatin treated mice, motor function was assessed using the rotarod apparatus (Med Associates INC, Georigia, VE). Briefly, mice were placed individually on the rotating system and trained for three days at 16 rpm with three trials per day. Each trial lasted for three minutes; on the third day all mice completed the 3 minute training session. In the actual motor function test, an accelerated rotarod assay (4–40 rpm over 5 min) was evaluated for three trails and the latency to fall was recorded. For details see supporting information section (File S2).

### Adhesive removal test for tactile hyposensitivity behavior

In order to examine the effect of cisplatin on sensory function, we used a modification of the adhesive removal test [22]. Briefly, a round adhesive patch (3/16” Teeny Touch-Spots, USA Scientific INC. Ocalo, FL) was placed on the plantar surface of the hind paws. The animal performance in a testing cage (20×20×13 cm^3^) was recorded in 15 min. The time until the mouse started to shake its paw or bring the paw to the mouth was recorded as a measure of the latency until the mouse noticed the presence of the adhesive patch on the paw. For details see supporting information section (File S1).

### Immunostaining for IENFs

For quantification of IENFs, 3×3 mm^2^ biopsies were dissected from the central plantar surface of the hind paws. Biopsies were immediately placed in Zamboni's fixative for 24 hrs, and transferred to 20% sucrose for at least 24 hrs, frozen in Optimal Cutting Temperature compound (OCT), and sliced into 25 µm sections. The sections were blocked for 2 hrs at room temperature in 0.1 M PBS containing 5% normal donkey serum/0.3% Triton X-100. The blocking solution was removed and the sections were incubated with an antibody against the pan neuronal marker PGP9.5 (AbD Serotec, Raleigh, NC; Rabbit, 1∶2000) along with anti-Collagen IV antibody (Southern Biotech, Birmingham, AL; Goat, 1∶100) at 4 °C overnight. Then the sections were washed in wash buffer (3 times one hour), and incubated with Alexa-594-donkey anti-rabbit (Life Technologies, Grand Island, NY 1∶500) and Alexa-488-donkey anti-goat (Invitrogen, Grand Island, NY 1∶500) at 4 °C overnight. Sections were washed in wash buffer and then three randomly chosen slices from each paw were quantified under a Leica fluorescence microscope. Nerve fibers that crossed the collagen stained dermal/epidermal junction into the epidermis were counted in three fields of each slice using the 40× objective. The length of the epidermis within each field was measured using ImageJ. IENF density was determined as the total number of fibers/length of epidermis (IENFs/mm). For details see supporting information section (File S4). Representative images captured by using In Cell 6000 microscope are shown.

### Statistical analysis

Data are expressed as mean ± SEM. Statistical analysis was carried out using repeated measure analysis of variance or dependent t test or one-way ANOVA followed by Bonferroni analysis. P<0.05 was considered statistically significant. Methods for calculating 50% withdrawal thresholds are detailed in file S5. Raw data are provided in the supporting information file S6.

## Results

### Effect of metformin on chemotherapy-induced neuropathic pain

**Figure 1:**
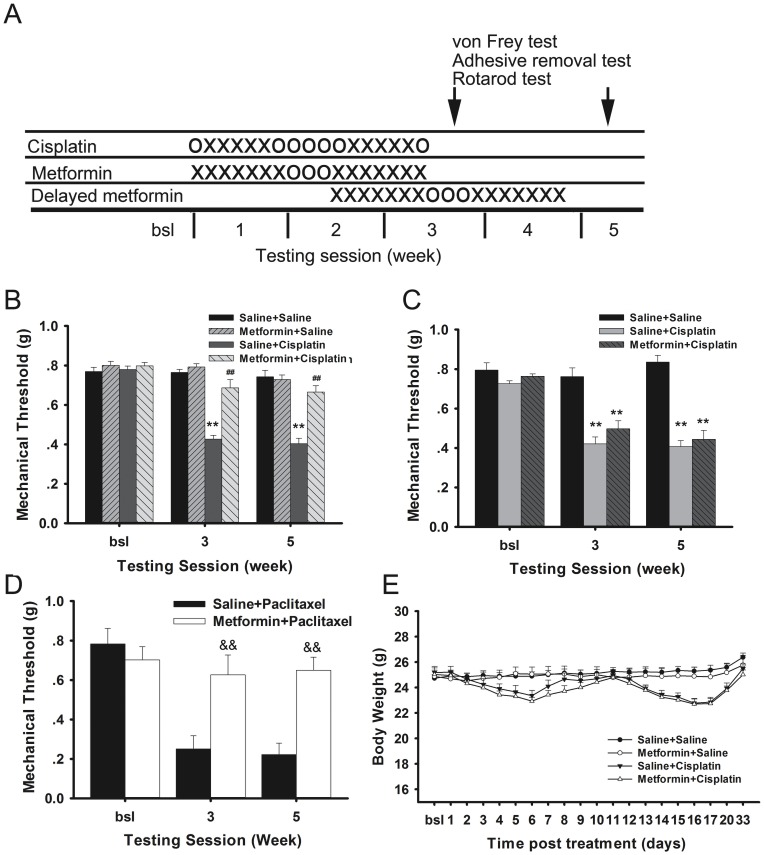
Effect of metformin on cisplatin- or paclitaxel-induced mechanical allodynia in mice. A). Treatment schedule; X: injection; O: no treatment. B) Mice (n = 10–12/group) were treated with cisplatin (cumulative dose 23 mg/kg i.p.) and metformin (200 mg/kg/dose i.p.) as depicted in panel A. Mechanical allodynia was quantified with von Frey hairs using the up and down method. C). Effect of delayed metformin treatment on cisplatin-induced mechanical allodynia. Mice (n = 8/group) received i.p. injections with cisplatin (cumulative dose 23 mg/kg i.p.) and delayed metformin (200 mg/kg/dose i.p.) as depicted in panel A and mechanical allodynia was monitored. D). Mice (n = 4–7/group) were treated with paclitaxel (10 mg/kg/every other day, i.p. for two weeks) and metformin (200 mg/kg i.p. daily from one day before until one day after paclitaxel) and mechanical allodynia was measured. E). Change in body weight after cisplatin and metformin treatment. ** p<0.01 vs. Saline+Saline group; ## p<0.01 vs. Saline+Cisplatin; && p<0.01 vs. Saline+Paclitaxel.

Mice were treated with two rounds of 5 daily i.p. injections with cisplatin with 5 days rest in between (Figure 1a). Figure 1b demonstrates cisplatin induced persistent mechanical allodynia measured at 3 and 5 weeks after the 1^st^ cisplatin injection. Metformin was administered at a dose that is used to treat diabetic mice (200 mg/kg daily) and that is efficacious in surgical nerve injury-induced neuropathic pain [4], for 2 rounds of 7 days, beginning 24 hours prior to the 1^st^ dose of cisplatin of each cycle. Co-administration of metformin treatment completely prevented cisplatin-induced mechanical allodynia (Figure 1b). Metformin treatment alone did not have any effect on mechanical sensitivity (Figure 1b).

To determine whether metformin treatment should be started together with cisplatin or can be delayed, we delayed metformin treatment until the 2^nd^ round of cisplatin treatment. The results in Figure 1c show that this delayed metformin treatment had no effect on mechanical allodynia. These results strongly suggested that concomitant but not delayed treatment with metformin prevents cisplatin-induced mechanical allodynia.

The beneficial effect of metformin was not limited to cisplatin-induced mechanical allodynia; also paclitaxel-induced mechanical allodynia was prevented by administration of metformin starting 24 hours prior to the first dose of paclitaxel (Figure 1d).

Metformin treatment did not affect body weight when given alone and did not have any effect on the transient loss of bodyweight in mice treated with cisplatin (Figure 1e) or paclitaxel (Data not shown).

### Cisplatin-induced sensory deficits and metformin treatment

**Figure 2:**
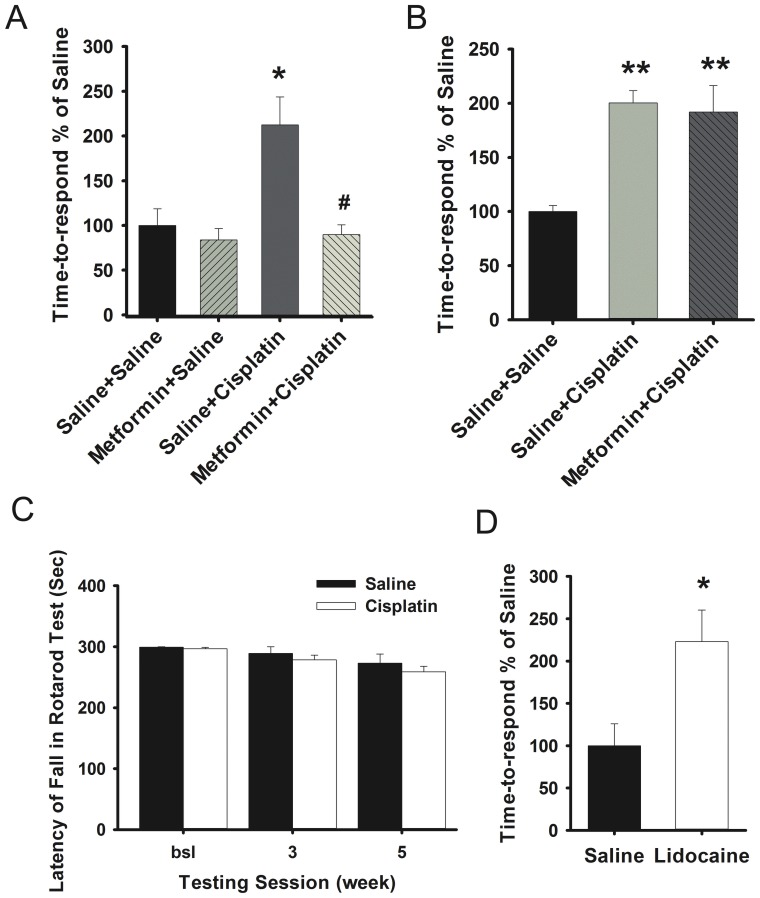
Effect of metformin treatment on cisplatin-induced sensory deficits. A). Mice (n = 6–10/group) were treated with cisplatin and metformin as described in Figure 1. The time to respond to an adhesive patch on the hind paw was monitored. B). Effect of delayed metformin on cisplatin induced sensory deficits (n = 7–8/group). C). Effect of cisplatin treatment on rotarod performance. Mice (n = 6/group) were treated with cisplatin and their performance on the rotarod was monitored as an index of motor coordination. E). Mice (n = 7/group) were treated with lidocaine (5 µL, 4% in saline) or saline and 10 mins. later, the time to respond to an adhesive patch on the hind paw was measured. * p<0.05, ** p<0.01 vs. Saline+Saline; # p<0.05 vs. Saline+Cisplatin.

The effect of cisplatin on the time-to-respond to an adhesive patch on the hind paw was recorded as a novel indicator of chemotherapy-induced sensory deficit. In cisplatin-treated mice the time-to-respond to the adhesive patch on the hind paw was significantly prolonged at 3 and 5 weeks after the 1^st^ cisplatin injection when compared to saline-treated mice (Figure 2a). Co-administration of metformin prevented the increase in latency to respond to the adhesive patch on the paw, whereas delayed treatment did not have any effect (Figure 2a, c). The time between the first response to the adhesive patch and subsequent removal of the patch was not affected by cisplatin treatment indicating normal motor function (data not shown). In addition, cisplatin-treated mice showed normal performance on a rotarod indicating that the prolonged time-to-respond to the patch on the hind paw cannot be attributed to potential cisplatin-induced motor function deficits (Figure 2d). As a positive control, the data in Figure 2e show that the time-to-respond to the adhesive patch was also significantly increased in lidocaine-treated mice. Collectively, our findings support the notion that the increase in time-to-respond to the adhesive patch in cisplatin-treated mice is related to a sensory deficit, which is prevented by metformin treatment.

### Protective effect of metformin on cisplatin-induced IENF loss

**Figure 3:**
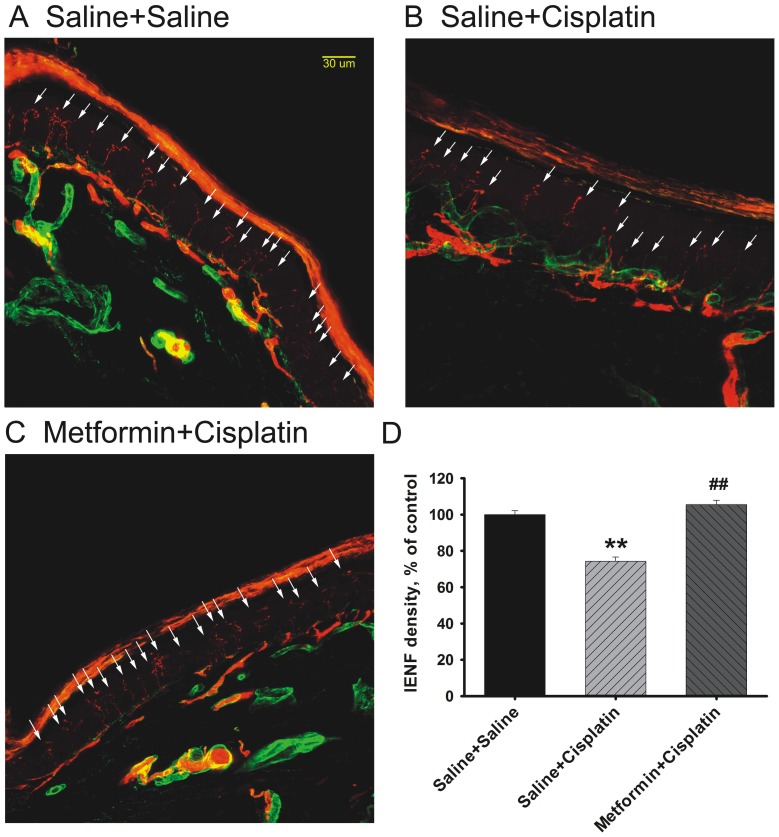
Effect of metformin on loss of intraepidermal nerve fibers induced by cisplatin. Mice (n =  4–6 per group) were treated with cisplatin and metformin as in figure 1. Paw biopsies obtained from the hind paw at 5 weeks after the start of treatment were stained for intraepidermal nerve fibers (PGP9.5; red) and collagen (green). (A) saline/saline; (B) saline/cisplatin; (C) metformin/cisplatin. White arrows indicate the intraepidermal nerve fibers stained by PGP9.5 (in red), which can clearly be seen crossing the basement membrane (in green) and extending as long lines into the epidermis. (D) Quantification of intraepidermal nerve fiber density. ** p<0.01 vs. Saline+Saline; ## p<0.01 vs. Saline+Cisplatin.

IENF loss had been reported in rat models of chemotherapy-induced neuropathy using cisplatin, oxaliplatin, taxol, paclitaxel and vincristine [13], [14], [23], [24]. We analyzed the effect of metformin treatment on the IENF density in hind and fore paw of cisplatin treated mice at 5 weeks after the first cisplatin treatment. Saline-treated mice showed abundant distribution of nerve fibers entering the epidermis. The IENF density was significantly decreased in response to cisplatin-treatment (Figure 3a–b). Notably, metformin significantly protected against this cisplatin-induced IENF loss (Figure 3c). There were no significant differences between metformin-treated cisplatin mice and saline- treated mice (Figure 3d).

## Discussion

The AMP kinase agonist metformin is an anti-diabetic drug that has been safely and widely used for the treatment of type 2 diabetes for decades. This is the first study to show that metformin protects against cisplatin- and paclitaxel-induced mechanical allodynia in a mouse model. Additionally, this is the first study to demonstrate a loss of sensory sensitivity associated with CIPN in a mouse model. We show that cisplatin increases the time to detect an adhesive patch on the hind paw and that metformin prevents development of this sign of sensory impairment or ‘numbness’ in cisplatin-treated mice. In search for an underlying mechanism, we show that metformin treatment protects against the loss of IENFs that occurs in response to cisplatin treatment. Collectively, these findings indicate that metformin protects against cisplatin-induced peripheral neuropathy by reducing peripheral nerve damage. We propose that these findings represent the discovery of a safe preventive treatment for chemotherapy-induced neuropathy. Because metformin is already widely used, rapid clinical translation of these findings should be possible.

Metformin is the first-line treatment for type II diabetes because of its insulin sensitizing effects [25]. Individuals with obesity or polycystic ovary syndrome are also frequently treated with metformin. In 2012, over 61 million prescriptions for metformin were filed in the United States [26]. There is evidence that metformin treatment of diabetics reduces the risk of cancer in this population. In addition, preclinical and clinical studies indicate that metformin treatment may increase the efficacy of cancer treatment. In a retrospective study in breast cancer patients with diabetes, patients treated with metformin had higher complete pathologic response rates to neoadjuvant therapy than diabetic patients treated with other antidiabetic drugs [27]. Similarly two retrospective cohort studies indicated that metformin treatment of diabetic patients with ovarian cancer may prolong disease specific and progression-free survival [28], [29], [30]. Preclinical data demonstrate that metformin inhibits cancer cell growth in vitro, enhances anti-tumor effects of chemotherapeutics in vitro, inhibits the inflammatory response associated with cancer and prolongs remission in response to chemotherapy in mouse xenograft models of cancer [31], [32], [33], [34]. Collectively these findings have given rise to an increasing number of clinical trials aimed at examining the efficacy of metformin in cancer treatment. The data we show here identify a potential additional beneficial effect of metformin in cancer treatment. Our finding that metformin protects against chemotherapy-induced peripheral neuropathy urges for inclusion of systematic assessment of neuropathy in trials with metformin in cancer patients.

Recent studies have shown that metformin alleviates pain amplification that occurs in response to peripheral nerve injury in mice and rats [4], [18]. In these surgical models of neuropathic pain, it was shown that metformin reverses already existing mechanical allodynia. We show here that metformin prevented development of cisplatin- and paclitaxel-induced mechanical allodynia only when treatment was started before start of the administration of these chemotherapeutics. In contrast to what was observed in traumatic models of neuropathy, we did not observe any beneficial effect of metformin when treatment was started after the first round of cisplatin treatment, when mechanical allodynia had already developed. This result is in contrast to previous observations in the spared nerve injury model in mice and spinal nerve ligation injury model in rats where metformin treatment after establishment of neuropathic allodynia effectively reversed this symptom of peripheral nerve injury. While the reasons for this discrepancy between models are not presently clear, there are several possible reasons. First, chemotherapy-induced neuropathy is mechanistically distinct from trauma-induced neuropathy. A therapeutic benefit of AMPK activation in trauma-induced neuropathy is related to decreases in mTOR and MAPK signaling in injured nerves [5], [35]. It is not currently known if these signaling pathways are altered in CIPN. On the other hand, CIPN involves clear changes in mitochondrial function that may be an important target for AMPK activation in CIPN [36]. If mitochondria are a relevant target for AMPK activation [37] in CIPN, it may not be possible to reverse this effect once established by the chemotherapeutic treatment. Another possibility is that while the effects of metformin in trauma-induced neuropathy are AMPK-mediated (this hypothesis is supported by similar findings with specific AMPK activating tool compounds), metformin effects in CIPN have a separate mechanism of action. This can be tested with future experiments with AMPK activating tool compounds. One AMPK-independent possibility is metformin-mediated blockade of organic cation transporter 2 [38] which is required for oxaliplatin-induced CIPN [39]. While these mechanism-based studies are undoubtedly important, the aim of these studies was to assess metformin as a potential treatment for CIPN and our studies give a clear rationale for preventative clinical trials with this drug.

At present, knowledge about the mechanisms underlying chemotherapy-induced sensory deficits remains limited. Although the pain component of CIPN has received the most attention, sensory deficits are often longer lasting and thus debilitating for patients in daily life. One of the aims of this study was to determine whether cisplatin treatment in mice leads to sensory impairment and to assess the effect of metformin on cisplatin-induced sensory impairment. To that end, we used the adhesive removal test, a sensitive method to evaluate both somatosensory and motor function in rats and mice [22]. In this test, an adhesive patch is placed on the plantar surface of the hind paw, and the latency to a behavioral response to the patch as well as latency to removal of the patch is assessed. The data show that the time to display a behavioral response to the adhesive patch placed on the paw is significantly prolonged in mice treated with cisplatin. This sign of cisplatin-induced ‘numbness’ was present without evidence of functional motor deficits. We also observed significant IENFs loss in mice treated with cisplatin. These findings are in line with earlier studies showing loss of IENFs in rats treated with chemotherapeutic agents such as paclitaxel [24], vincristine [23], oxaliplatin [13] and cisplatin [14]. In humans exposed to chemotherapy, damage to peripheral nerves in the extremities has also been reported [1]. Moreover, IENFs transmit sensations of tactile, cold, thermal and mechanical origin from the skin, and loss of IENFs has been shown to correspond to loss of these sensations in humans [40]. We show here that mice treated with cisplatin display sensory deficits and los of IENFs in the hind paws consistent with results using the adhesive tactile test. Co-administration of metformin prevented both sensory deficits and loss of IENFs in the paws. These results strongly suggest that the cisplatin-induced of loss of IENFs in the paw contributes to chemotherapy-induced sensory deficits. The protective effect of metformin on loss of IENFs is consistent with earlier reported neuroprotective activity of metformin in models of brain damage. Moreover, in a model of peripheral nerve damage-induced neuropathy it has been shown that metformin increases the level of ApoE, a 34 kDa glycoprotein that is a major determinant of lipid transport and metabolism [18]. In addition, ApoE plays an important role in peripheral and central neuroregeneration and remyelination [18], [41], [42], [43]. It remains to be determined whether metformin-induced increases in ApoE also contribute to the protective effect of metformin in our mouse model of chemotherapy-induced peripheral neuropathic pain.

It may seem contradictory that chemotherapy-induced decrease in IENF density is associated with decreased sensory function, but enhanced pain sensitivity. Electrophysiological and biochemical studies in cisplatin-treated rats show that sensitization of sensory neurons, increased spontaneous activity and prolonged after discharges in response to stimulation, plays a key role in pathological pain behaviors following platinum drugs [44]. This hyperresponsiveness of sensory neurons may be due to up-regulation of a variety of channels in DRG neurons and/or changes in signaling leading to enhanced activity in these channels following cisplatin treatment [19]. While the mechanism of action of metformin has not been elucidated in these studies, the present findings are consistent with an AMPK-mediated mechanism of action because activation of this kinase dampens signaling in pathways linked to sensory neuron hyperexcitability and may modulate the expression of channels via transcription and/or translation regulation downstream of AMPK activation [4], [5].

In conclusion, this study is the first to demonstrate that metformin protects against chemotherapy-induced neuropathic pain and numbness in a mouse model. The beneficial effect of metformin is associated with a reduction in the loss of plantar IENFs. Whether these neuroprotective effects of metformin treatment in the mouse model of cisplatin-induced peripheral neuropathy are the direct cause of the protective effect of metformin treatment on pain and numbness need to be further investigated. There are no FDA-approved drugs to treat CIPN. As a result of CIPN a significant subset of patients receives sub-optimal doses of anti-cancer treatment. Moreover, CIPN markedly reduces quality of life both during and after completion of treatment.

Because metformin has already been widely used, these findings strongly argue for an immediately available novel treatment to prevent chemotherapy-induced peripheral neuropathy in patients treated for cancer. The ongoing studies on the usefulness of metformin as an add-on therapy in cancer treatment should include analysis of the potential beneficial effect of metformin on chemotherapy-induced neuropathy as an outcome measure.

## Supporting Information

File S1 **Von Frey test for mechanical allodynia.**
(DOCX)

File S2 **Rotarod test for motor impairment.**
(DOCX)

File S3 **Adhesive Removal Test.**
(DOC)

File S4 **Protocol Immunohistochemistry of intraepidermal nerve fibers.**
(DOC)

File S5 **Excell sheet for calculation of 50% withdrawal threshold.**
(XLS)

File S6 **Raw data.**
(XLSX)
