# Supplementary material for: The Anti-Diabetic Drug Metformin Protects against Chemotherapy-Induced Peripheral Neuropathy in a Mouse Model
Source: PLoS One. 2014 Jun 23;9(6):e100701. doi: 10.1371/journal.pone.0100701 (PMC4067328; doi:10.1371/journal.pone.0100701)
Supplement: File S1 — Von Frey test for mechanical allodynia. (DOCX) [file pone.0100701.s001.docx]

**Supporting information file S1: Von Frey test for mechanical allodynia**

Mechanical allodynia was measured as the hind paw withdrawal response to von Frey hair stimulation using the up-and-down method as we described previously [1].

PROTOCOL:

1. Make sure that the equipment set is complete;

a. Mesh stand (IITC; <http://www.iitcinc.com/Mesh_Stand.html> )

b. Animal enclosures with lid (10 x 10 x 13 cm3).

c. von Frey hair (0.02, 0.07, 0.16, 0.4, 0.6, 1.0 and 1.4 g) (Stoelting, Wood Dale, Illinois, USA)

2. Training session: the animal was placed into the plastic enclosure on the mesh stand for 1 hour/day for 3 days before the testing.

3. Testing session: the animal is placed into the plastic enclosure on the mesh stand for acclimation for 30 mins prior to testing.

4. A trial began with the application of the 0.16 g hair.

5. A positive response was defined as a clear paw withdrawal or shaking, marked with “X”; otherwise negative response marked with “O”.

6. Whenever a positive response occurred, the next lower hair was applied, and whenever a negative response occurred, the next higher hair was applied.

7. The testing consisted of five more stimuli after the first change in response occurred.

8. The pattern of response marked with “O”“X” was converted to a 50% von Frey threshold using an excel formula (see the attached excel file) according to the method described previously [2].

9. Baseline measures were taken on three days prior to the start of injections and the average is presented as baseline in the graph.

References：

1. Wang H, Heijnen CJ, Eijkelkamp N, Garza Carbajal A, Schedlowski M, et al. (2011) GRK2 in sensory neurons regulates epinephrine-induced signalling and duration of mechanical hyperalgesia. Pain 152: 1649-1658.
2. Chaplan SR, Bach FW, Pogrel JW, Chung JM, Yaksh TL (1994) Quantitative assessment of tactile allodynia in the rat paw. Journal of neuroscience methods 53: 55-63.
