# Supplementary material for: The Anti-Diabetic Drug Metformin Protects against Chemotherapy-Induced Peripheral Neuropathy in a Mouse Model
Source: PLoS One. 2014 Jun 23;9(6):e100701. doi: 10.1371/journal.pone.0100701 (PMC4067328; doi:10.1371/journal.pone.0100701)
Supplement: File S2 — Rotarod test for motor impairment. (DOCX) [file pone.0100701.s002.docx]

**Supporting information file S2: Rotarod test for motor impairment**

To control for the possibility that behavioral changes are due to the motor impairment in cisplatin treated mice, motor function was assessed using the rotarod apparatus (Med Associates INC, Georigia, Vermont).

1. Training: Mice are placed individually on the rotating system and trained for three days at 16 rpm with three trials per day for three days. Each trial lasted for three minutes with 5 mins’ interval between two trials.
2. Test: In the actual motor function test, an accelerated rotarod assay (increasing speed linearly from 4-40 rpm over 5 min) is evaluated in three trials on each test day and the latency to fall was recorded. The mean value of three trials was defined as the final latency to fall.
