# Supplementary material for: The Anti-Diabetic Drug Metformin Protects against Chemotherapy-Induced Peripheral Neuropathy in a Mouse Model
Source: PLoS One. 2014 Jun 23;9(6):e100701. doi: 10.1371/journal.pone.0100701 (PMC4067328; doi:10.1371/journal.pone.0100701)
Supplement: File S3 — Adhesive Removal Test. (DOC) [file pone.0100701.s003.doc]

Supportive information file S3: Adhesive Removal Test

1. Make sure that the equipment is set up;
   1. Mirror placement below the cage so that the paws can be seen optimally
   2. Large plastic cage (20 x 20 X 13 cm^3^) on a stand so that the bottom can be seen.
   3. Timer
   4. Stickers (3/16’’ Teeny Touch-Spots, Diversified Biotech)
2. Training session: the animal was placed into the plastic cage for 15 mins acclimation one day before the testing.
3. Testing session: Place the sticker on one hindpaw, put the animal in the large cage and record the time until the animal shakes its paw or brings the paw to the mouth as a measure of the latency until the mouse noticed the presence of the adhesive patch on the paw. Maximal time: 15 min.
4. The latency to fully remove the sticker is also recorded.
5. If animals exceed the 15 min time-limit without sticker removal, remove the sticker yourself and place the next sticker.
6. After testing animal #1 put animal back in cage with the remainder of test-subjects & remove the defecation. Wipe the interior of the cage with a tissue in between subjects. Clean the cage with water/ethanol after you have finished the entire session.
7. Repeat steps 3-6 for the following animals.
