# Supplementary material for: The Anti-Diabetic Drug Metformin Protects against Chemotherapy-Induced Peripheral Neuropathy in a Mouse Model
Source: PLoS One. 2014 Jun 23;9(6):e100701. doi: 10.1371/journal.pone.0100701 (PMC4067328; doi:10.1371/journal.pone.0100701)
Supplement: File S4 — Protocol Immunohistochemistry of intraepidermal nerve fibers. (DOC) [file pone.0100701.s004.doc]

**Supporting information file S4**: **Protocol Immunohistochemistry of intraepidermal nerve fibers**

Staining:

1. Mice are overdosed with pentobarbital and a 3-mm biopsy is taken.

2. The biopsies are placed in Zamboni’s fixative, left overnight at 4ºC.

3. Tissue is then transferred to 20% sucrose for at least 24 hr.

4. Tissue is frozen in OCT and sliced into 25 μm free-floating sections.

5. The slices are blocked by 0.1M PBS with 0.3% Triton X-100 + 5% normal donkey serum, 2 hr, RT.

6. The slices are incubated with primary Ab overnight at 4ºC.

PGP9.5 (AbD Serotec, Cat. No. 7863-0504, rabbit), 1:2000

Collagen IV, (Southern Biotech, Cat. No. 1340-01, goat)1:100

7. The slices are washed 3 times for 1 hr each in PBST.

8. Secondary Ab are added for an overnight incubation at 4ºC.

594-Donkey anti-rabbit, 1:500

488-Donkey anti-goat, 1:500

9. The slices are washed 3 times for 1hr each in PBST, then 30 min in PBS

10. Mount the slices onto slides.

Counting:

1. Randomly choose 3 slices from each animal.

2. For each slices, take 3 fields, count the numbers of nerve fiber that cross the dermal/epidermal junction into the epidermis, using a 40* objective.

3. Measure the length of the epidermis within each fields.

4. IENF density=step2/step3 (IENF/mm).

5. Relative IENFs density %= 100* IENFs density of testing group/ IENFs density of saline group %
